# Peculiarities of the first time diagnosed mental disorders formationin after the coronavirus disease COVID-19

**DOI:** 10.1192/j.eurpsy.2024.1078

**Published:** 2024-08-27

**Authors:** N. O. Maruta, V. Y. Fedchenko, T. V. Panko, I. O. Yavdak

**Affiliations:** Borderline psychiatry, “Institute of Neurology, Psychiatry and Narcology of the NAMS of Ukraine” SI, Kharkiv, Ukraine

## Abstract

**Introduction:**

The world community is only at the beginning of awareness of the peculiarities of the formation, course and outcome of the psychopathological consequences of the impact of the SARS-CoV-2 pandemic.

**Objectives:**

To investigate the clinical and anamnestic features and their influence on the formation of psychopathological consequences in patients with first diagnosed mental disorders who have experienced COVID-19 and were exposed to the stressors of the SARS-CoV-2 pandemic.

**Methods:**

97 patients with first diagnosed mental disorders who have experienced COVID-19 and were exposed to the stressors of the SARS-CoV-2 pandemic were examined (F 32.0-32.2 – 34 patients, F 40-45 – 32 patients, F 06.3-06.6 – 31 patients). Clinical-psychopathological, clinical-amnestic methods, including information about the experienced coronavirus disease COVID-19, the impact of the stressors of the SARS-COV-2 pandemic, and methods of statistical analysis were applied.

**Results:**

The conducted research made it possible to identify the phenomenological structure of mental disorders that develop after the coronavirus disease COVID-19. This structure includes depressive disorders (35.05%), neurotic, stress-related and somatoform disorders (32.99%), as well as mental disorders of organic genesis (31.96%). An important result of the study was the determination of the heterogeneity of mental pathology in the context of the influence of stressogenic factors of the pandemic and other psychogenies. In this aspect, all mental and behavioral disorders must be divided into 3 variants of pathology, which differ in the mechanisms of formation: caused by the pathoplastic factors of COVID-19 and the patient’s personal reactions to the disease; related to the psychogenic effects of the stressors of the SARS-COV-2 pandemic; with a combined mechanism of influence of pathoplastic and psychogenic factors. Certain diagnostic and phenomenological regularities characteristic of each of the options are defined. The influence of pathoplastic factors and personal reactions to the disease is associated with the formation of depressive disorders. Pandemic stressors most often cause the development of neurotic, stress-related and somatoform disorders. Under the influence of combined factors, disorders of organic genesis are formed to a greater extent. The initial manifestations of pathology also differ with different formation mechanisms: when pathoplastic factors predominate, asthenia, depression and sleep disturbances prevail; with leading psychogenic influences – anxiety and tension; when the above factors are combined - asthenia, stress and cognitive disorders.

**Conclusions:**

The significance of the obtained data lies in the possibility of studying the role of the psychopathological consequences of COVID-19 in the genesis of mental disorders.

**Disclosure of Interest:**

None Declared

